# Biocontrol Efficacy of Endophyte *Pseudomonas poae* to Alleviate *Fusarium* Seedling Blight by Refining the Morpho-Physiological Attributes of Wheat

**DOI:** 10.3390/plants12122277

**Published:** 2023-06-12

**Authors:** Ezzeldin Ibrahim, Raghda Nasser, Rahila Hafeez, Solabomi Olaitan Ogunyemi, Yasmine Abdallah, Arif Ali Khattak, Linfei Shou, Yang Zhang, Temoor Ahmed, Ashraf Atef Hatamleh, Munirah Abdullah Al-Dosary, Hayssam M. Ali, Jinyan Luo, Bin Li

**Affiliations:** 1 State Key Laboratory of Rice Biology and Breeding, Ministry of Agriculture Key Laboratory of Molecular Biology of Crop Pathogens and Insects, Key Laboratory of Biology of Crop Pathogens and Insects of Zhejiang Province, Institute of Biotechnology, Zhejiang University, Hangzhou 310058, China; ezzelbehery8818@yahoo.com (E.I.); rahila.impp@gmail.com (R.H.); sollybombom@yahoo.com (S.O.O.); yasmeen.abdallah@mu.edu.eg (Y.A.); arifalikh2020@hotmail.com (A.A.K.); taloyament@163.com (Y.Z.); temoorahmed@zju.edu.cn (T.A.); 2 Department of Vegetable Diseases Research, Plant Pathology Research Institute, Agriculture Research Centre, Giza 12916, Egypt; 3 Ministry of Agriculture Key Laboratory of Molecular Biology of Crop Pathogens and Insect Pests, Institute of Insect Sciences, College of Agriculture and Biotechnology, Zhejiang University, Hangzhou 310058, China; raghdanasser@mu.edu.eg; 4 Zoology and Entomology Department, Faculty of Science, Minia University, Elminya 61519, Egypt; 5 Station for the Plant Protection & Quarantine and Control of Agrochemicals Zhejiang Province, Hangzhou 310004, China; lfshou@163.com; 6 Department of Botany and Microbiology, College of Science, King Saud University, Riyadh 11451, Saudi Arabia; ahatamleh@ksu.edu.sa (A.A.H.); almonerah@ksu.edu.sa (M.A.A.-D.); hayhassan@ksu.edu.sa (H.M.A.); 7 Department of Plant Quarantine, Shanghai Extension and Service Center of Agriculture Technology, Shanghai 201103, China

**Keywords:** cell-free supernatants, deoxynivalenol, endophytes bacteria, enzymatic activities, *F. graminearum*

## Abstract

Some endophyte bacteria can improve plant growth and suppress plant diseases. However, little is known about the potential of endophytes bacteria to promote wheat growth and suppress the *Fusarium* seedling blight pathogen *Fusarium graminearum*. This study was conducted to isolate and identify endophytic bacteria and evaluate their efficacy for the plant growth promotion and disease suppression of *Fusarium* seedling blight (FSB) in wheat. The *Pseudomonas poae* strain CO showed strong antifungal activity in vitro and under greenhouse conditions against *F. graminearum* strain PH-1. The cell-free supernatants (CFSs) of *P. poae* strain CO were able to inhibit the mycelium growth, the number of colonies forming, spore germination, germ tube length, and the mycotoxin production of FSB with an inhibition rate of 87.00, 62.25, 51.33, 69.29, and 71.08%, respectively, with the highest concentration of CFSs. The results indicated that *P. poae* exhibited multifarious antifungal properties, such as the production of hydrolytic enzymes, siderophores, and lipopeptides. In addition, compared to untreated seeds, wheat plants treated with the strain showed significant growth rates, where root and shoot length increased by about 33% and the weight of fresh roots, fresh shoots, dry roots, and dry shoots by 50%. In addition, the strain produced high levels of indole-3-acetic acid, phosphate solubilization, and nitrogen fixation. Finally, the strain demonstrated strong antagonistic properties as well as a variety of plant growth-promoting properties. Thus, this result suggest that this strain could be used as an alternate to synthetic chemicals, which can serve as an effective method of protecting wheat from fungal infection.

## 1. Introduction

Wheat (*Triticum aestivum* L.) is the third-largest food crop after maize and rice [1]. *F. graminearum* (Fg) is one of the common fungal diseases that threatens many economic crops, such as wheat, rice, barley, and maize [2,3]. The fungus causes growth and yield losses in wheat by causing *Fusarium* seedling blight (FSB) and *Fusarium* head blight (FHB) [4]. FSB can cause severe damage to developing wheat seedlings in the growing season through pre- and post-emergence damping-off, coleoptiles blight, and leaf blight. Moreover, the fungus can attack wheat spikelets, causing a significant loss in yield production [5,6]. In addition to yield loss, the fungus produces several mycotoxins, such as zearalenone (ZEN) and deoxynivalenol (DON), which can have adverse health effects on the organisms that consume them [7,8]. Despite severe economic and health concerns, FSB-resistant wheat varieties are becoming rare, and chemicals are the only options for controlling this fungus. However, there are several issues with fungicide applications. First, *F. graminearum* invades wheat plants in different growth stages, and control with chemicals is very expensive. Second, during the infection of wheat plants during the flowering period, which coincides with the rains in summer, the pesticides are washed off with rainwater, and therefore their effectiveness is very weak. Third, the irregular and multiple applications of fungicides cause several adverse impacts and dangers, such as the growth of fungicide-resistant strains, the killing of helpful microbes in the environment, a decline in biodiversity, and a toxic effect on human health [9,10]. Nowadays, biological control through the use of plant growth-promoting bacteria (PGPB) is appropriately considered as an alternative route for plant disease management [10,11]. Plants are naturally associated with diverse microorganisms in various ways. A group of these microorganisms, endophytic bacteria, can colonize the internal part of plant tissue and establish a mutual symbiosis relationship [12,13]. Many endophyte bacteria have been reported to combat phytopathogens, induce plant growth promotion, and diminish environmental stress [13,14]. Endophytic bacteria employ several mechanisms in controlling plant diseases. For example, antibiosis and competition for nutrients can directly inhibit plant pathogens, while induced plant systematic resistance indirectly halts the infection of pathogens in plants [15]. Furthermore, endophytic bacteria collectively play an important role to enhance seedling emergence and plant growth promotion, increase nutrient availability uptake for plants, and enhance host resistance to both biotic and abiotic stress [16,17]. *Pseudomonas* spp. is one among the endophytic bacteria, and it is the most efficient biocontrol agent. *Pseudomonas* spp. can survive and adapt to complex environments and produce a wide range of secondary metabolites that play an important role in protecting plants from microbial infection while promoting their growth [18,19]. However, the effect of endophytic bacteria in inhibiting phytopathogenic fungi, especially *Fusarium* seedling blight, has not been adequately studied so far. Therefore, the aim of this study was to isolate, identify, and characterize endophytic bacteria and study their properties as an antifungal agent against *F. graminearum,* and explore their role in promoting the growth of wheat plants. 

## 2. Results

### 2.1. In Vitro Antifungal Activity and Identification of the Endophytic Bacterium

**Figure 1:**
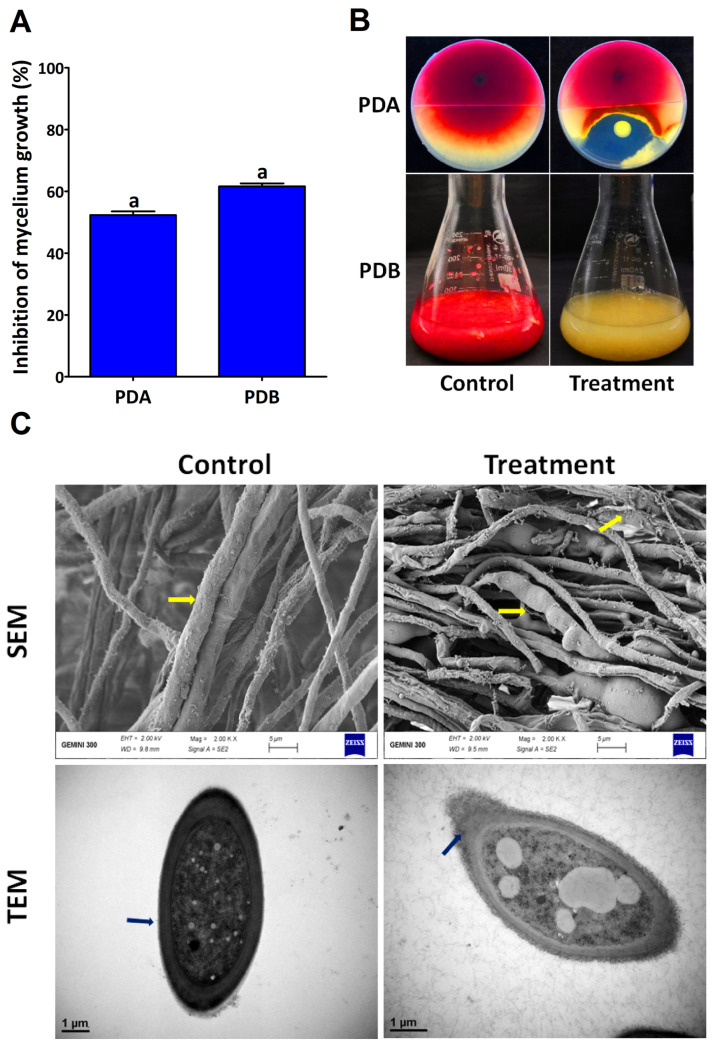
In vitro antifungal activity of the endophytic bacterium *P. poae* strain CO against *F. graminearum* strain PH-1 (**A**). The inhibition of mycelium growth in potato dextrose agar (PDA) and potato dextrose broth (PDB) mediums (**B**). TEM and SEM images of Fg strain PH-1 treated and untreated with endophytic bacterium strain CO (**C**). Data are mean value ± standard error of three replicates, and bars with the same letters are not significantly different in the LSD test (*p* < 0.05).

Seven endophytic bacterial strains were isolated from the roots and leaves of garlic. One of them, namely CO in PDA and PDB media, showed strong antagonistic effects on *F. graminearum* (Figure 1A,B). Notably, the relative fungal growth of PH-1 was inhibited by endophytic bacteria at a rate of 51.11% after five days of incubation in the PDA medium, while it was 65.70% in the PDB medium. Moreover, PH-1 hyphal were collected from the periphery of colonies and examined with SEM and TEM microscopes. The micrographs revealed that the hyphal of the control plates were typical and branched, with thick cell walls (Figure 1C). In contrast, the hyphal in the periphery culture plates of strain CO were abnormal, swollen, and damaged, with broken and destructed cell walls.

**Figure 2:**
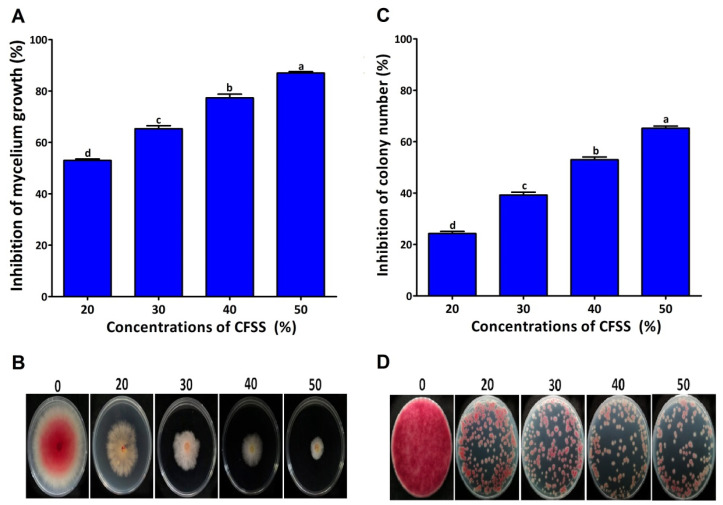
In vitro antifungal activity of CFSs at different concentrations against *F. graminearum* strain PH-1. The inhibition of mycelium growth (**A**,**B**) and the inhibition of colony number (**C**,**D**). Data are mean value ± standard error of three replicates, and bars with the different letters are significantly different in the LSD test (*p* < 0.05).

The results of the effect of different concentrations of CFSs of endophytic bacteria on the mycelial growth of *F. graminearum* under in vitro conditions revealed that the different concentrations of CFSs (20%, 30%, 40%, and 50% *v*/*v*) inhibited the mycelial growth of *F. graminearum* at the rates of 53.00%, 65.33%, 77.33%, and 87.00% (Figure 2A,B). The colony numbers were counted after three days of incubation on a PDA medium supplemented with CFSs. Different concentrations of CFSs (20%, 30%, 40%, and 50% *v*/*v*) showed different levels of inhibition of *F. graminearum* colony counts (Figure 2C,D). The colony counts were reduced at all concentrations compared to the control, where the highest inhibition was 65.25% in PDA treated with 50% CFSs. 

**Figure 3:**
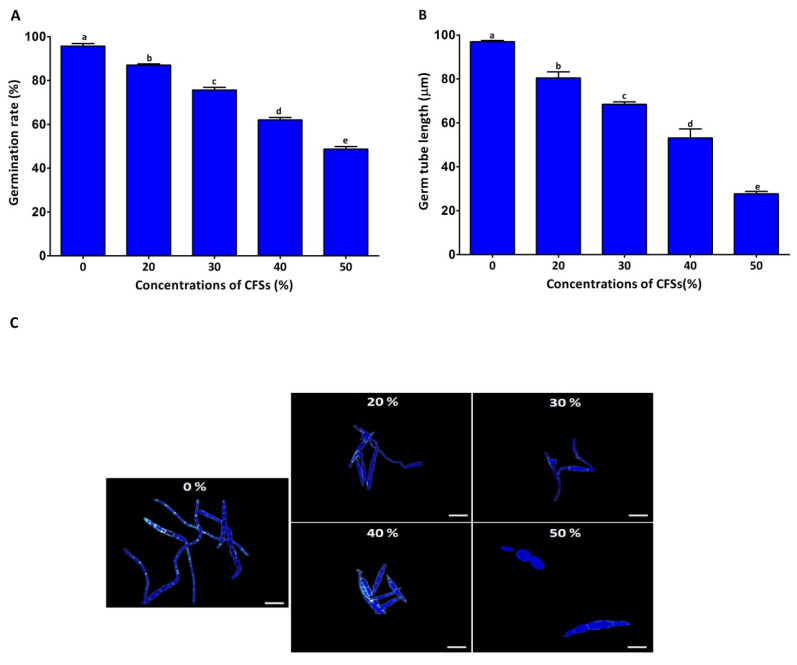
In vitro antifungal activity of CFSs at different concentrations against spore germination and germ tube length of *F. graminearum* strain PH-1. Inhibition of spore germination (**A**), inhibition of germ tube length (**B**), and light microscope images of spores treated with different concentrations of CFSs (**C**). Data are mean value ± standard error of three replicates, and bars with the different letters are significantly different in the LSD test (*p* < 0.05).

**Figure 4:**
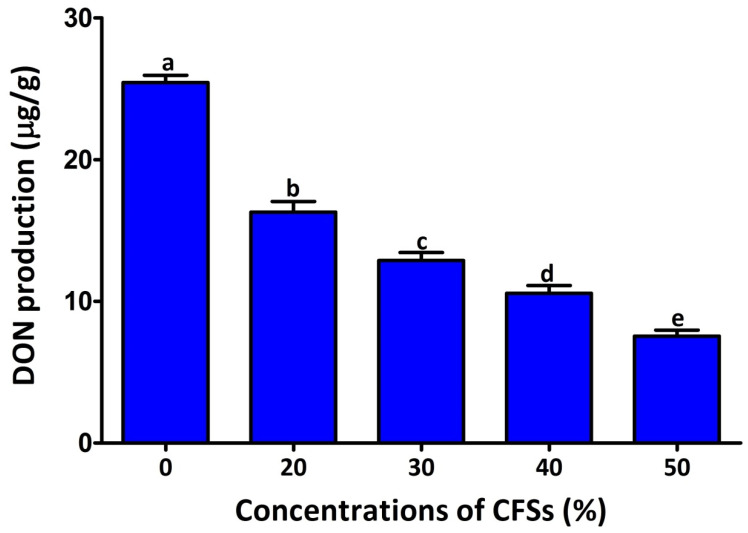
Effect of the CFSs at different concentrations on deoxynivalenol (DON) production. Data are mean value ± standard error of three replicates, and bars with the different letters are significantly different in the LSD test (*p* < 0.05).

Spore germination is known to be the first stage of fungal mycelium development. Understanding the inhibitory activity of CFSs on spore germination would help confirm the fungus’s inhibitory activity. Therefore, the effect of different concentrations of CFSs on *F. graminearum* spore germination and germ tube length was recorded after seven hours (Figure 3). Consistently, all the different concentrations of CFSs (20, 30, 40, and 50% *v*/*v*) exhibited inhibition by 13.00%, 14.44%, 38.93%, and 51.33%, respectively, of the spore germination rate, while the inhibition rate of the germ tube length was 16.57%, 28.57%, 43.86%, and 69.29%, respectively. The findings of this study demonstrated that CFSs significantly reduced the DON production of *F. graminearum* strain PH-1 in the GYEP medium (Figure 4). The DON production rate of strain PH-1 was 25.45 μg/g in the absence of CFSs. In contrast, the DON production rates were 16.30, 12.89, 10.56, and 7.54 μg/g in the presence of the CFSs at 20%, 30%, 45%, and 50% (*v*/*v*), respectively. The findings of this study points to the significant potential of CFSs in lessen DON contamination in cereals contaminated with *F. graminearum*.

**Figure 5:**
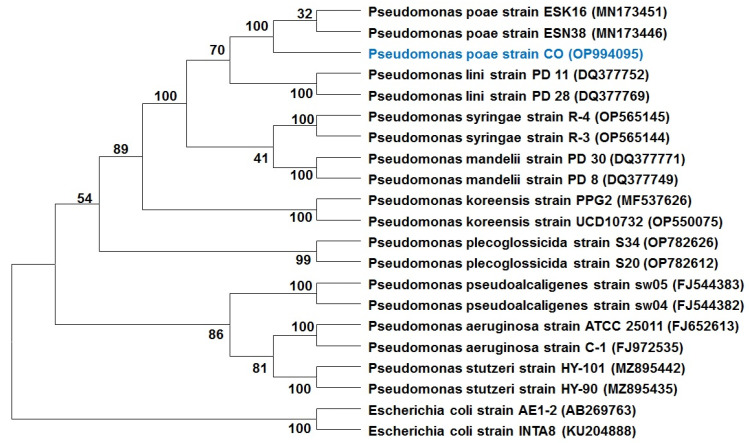
Phylogenetic tree of the endophytic bacterium *P. poae* strain CO isolated from garlic leaves, constructed using 16S rRNA gene sequences.

As a result, this isolate was subjected to molecular identification. Gene sequence analysis of endophytic strain was performed using the 16S rRNA gene. The resulting sequence was aligned using BLAST (the basic local alignment search tool) from the NCBI (National Center for Biotechnology Information) website (https://www.ncbi.nlm.nih.gov/BLAST accessed on 28 December 2022). BLAST results revealed that the CO 16S rDNA sequence is highly homologous to sequences belonging to *Pseudomonas* species. The 16S rDNA sequences of CO and their homologous sequences were then collected and used to construct a phylogenetic tree according to genetic distance. Phylogenetic analysis indicated that CO was classified as *Pseudomonas poae* (Figure 5). 

### 2.2. Suppression of FSB under Greenhouse Conditions 

**Figure 6:**
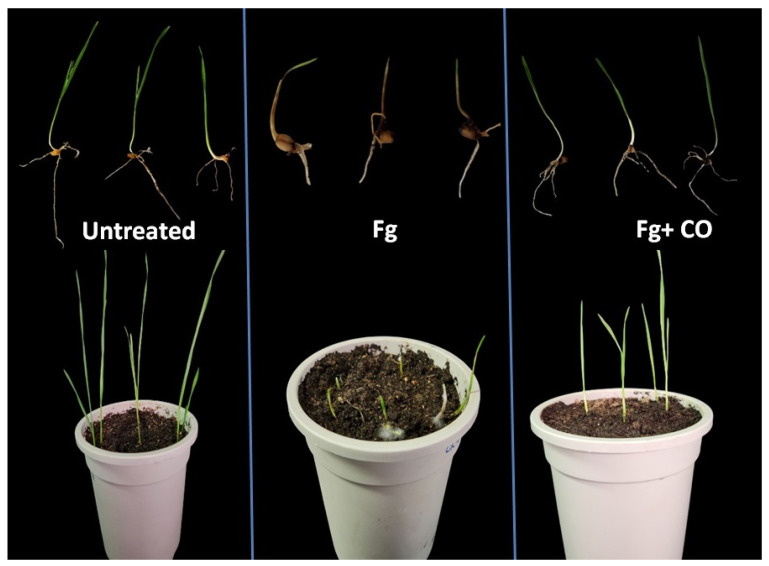
Inhibition of FSB under greenhouse condition using endophytic bacterium. The *P. poae* strain CO enhanced seedling-emergence growth in infected soil experimentally.

In this study, biocontrol experiments were performed to evaluate the bioprotection of the endophytic bacterium CO strain against FSB caused by *F. graminearum*. The entophytic bacterium *P. poae* enhanced seedling emergence from seeds planted in soil experimentally infected with a fungus inoculum producing seedling blight disease, compared to the group planted in fungus-infested soil without the bacterium (Table 1 and Figure 6). The treatment of wheat seeds with endophytic bacteria reduced the symptoms of seedling blight. In the presence of *F. graminearum*, the emergence rate was 15%, but when the bacteria were added, the emergence rate increased significantly to 70%. In addition, CO-treated seeds showed lower rates of pre- and post-emergence damping-off compared to non-CO-treated seeds sown in Fg-infected soil. Pre- and post-emergence damping-off rates for non-CO-treated seeds were 85.00% and 80.33%, respectively, whereas for treated seeds these rates were 30.00% and 7.10%. Moreover, the root length, shoot length, fresh weight, dry weight, seedling length vigor index, and seedling weight vigor index were significantly increased in CO-treated seedlings compared to the seeds planted in fungus-infested soil without treatment. 

**Figure 7:**
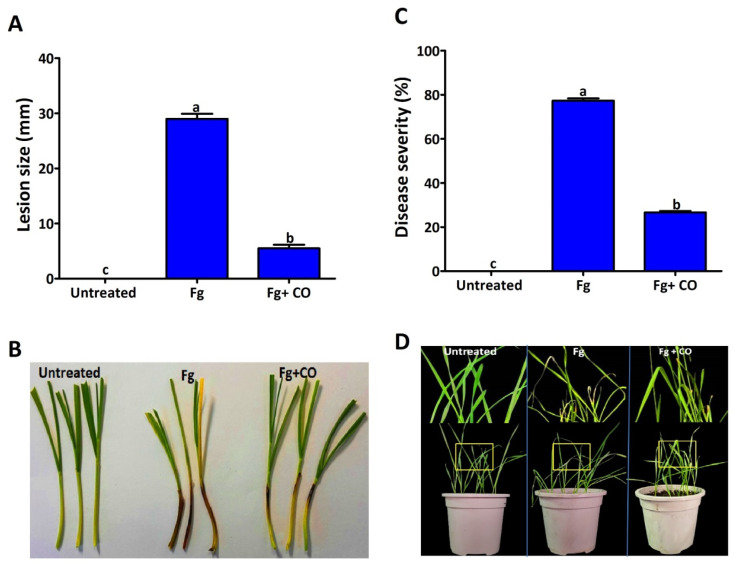
Protection of seedlings by the endophytic bacterium P. *poae* strain CO against FSB. The lesion size 7 days after inoculation was reduced in the coleoptiles of seedlings treated with Fg + *P. poae* compared to coleoptiles treated with Fg (**A**,**B**). Disease severity was reduced in the leaves of seedlings treated with Fg + *P. poae* compared to leaves treated with Fg (**C**,**D**). Data are mean value ± standard error of three replicates, and bars with the different letters are significantly different in the LSD test (*p* < 0.05).

In addition, the antifungal activity of *P. poae* strain CO was tested by measuring coleoptiles infection. In the positive group (treated with Fg), after 7 dpi, the dark brown lesion almost covered the coleoptile area (about 3 cm), as shown in Figure 7A,B. At the same time, the treatment with CO effectively reduced the pathogenicity of Fg, as the lesion size was 0.5 cm, while the control treatment (untreated) showed no symptoms. In addition to the tests for infection of wheat seeds and Coleoptiles, the antifungal activity of *P. poae* strain CO was also tested by measuring the infection of leaves. The data obtained showed that the severity of seedling blight was reduced in leaves treated with the endophytic strain compared to the control (Figure 7C,D). Disease severity was 76% for leaves treated with Fg compared to 15% for leaves treated with Fg mixed with CO.

### 2.3. Antifungal Mechanism 

**Figure 8:**
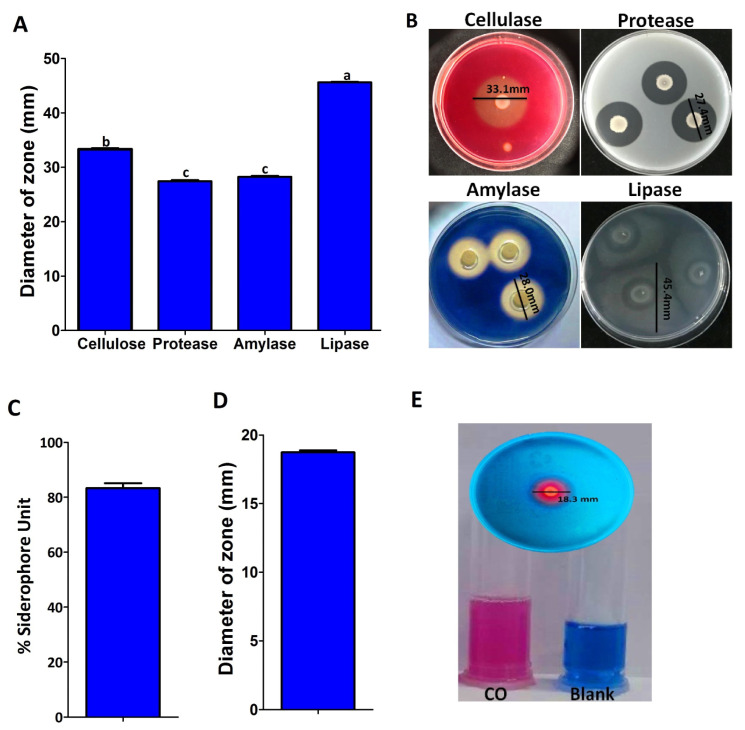
Detection of enzymatic activity and siderophore production of endophytic bacterium *P. poae* stain CO; zone diameter (mm) of cellulase, protease, amylase, and lipase of strain CO (**A**); colonies of *P. poae* surrounded by zones of extracellular enzymatic activity in the Petri dishes (**B**); siderophores unit (**C**); siderophores diameter (mm) (**D**); and the endophytic bacterium strain CO changed the CAS color from blue to purple, indicating its ability to produce siderophore (**E**). Data are mean value ± standard error of three replicates, and bars with the same letters are not significantly different in the LSD test (*p* < 0.05).

**Figure 9:**
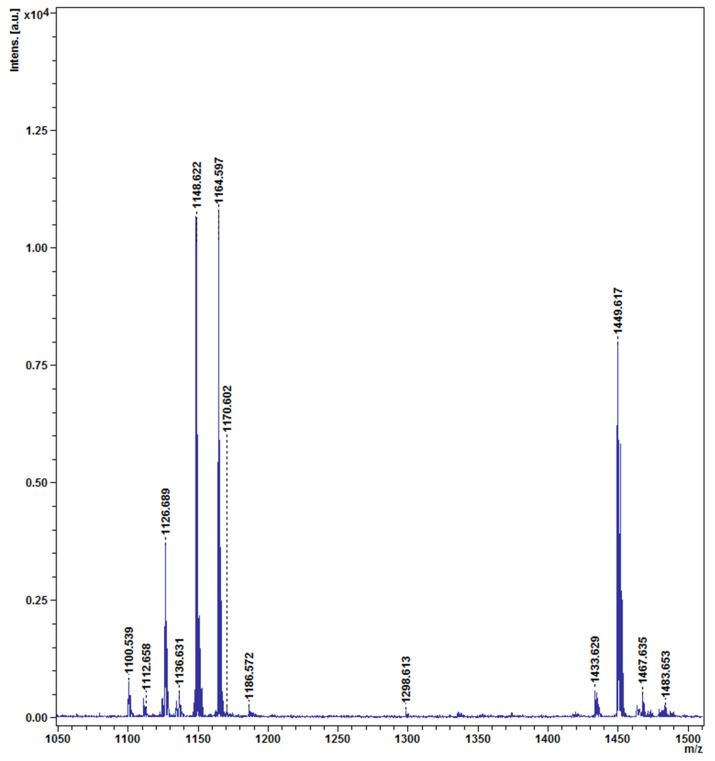
Detection of metabolites produced by endophytic bacterium *P.poae* strain CO using MALDI—TOF MS.

According to the results of hydrolytic enzymes, the CO strain was capable of producing cellulase, protease, amylase, and lipase enzymes (Figure 8A,B). When the endophytic bacterium was inoculated on carboxymethyl cellulose medium, skim milk agar medium, starch agar medium, and basal medium supplemented with Tween 80 (1% *v*/*v*), the zone sizes around the CO colonies reached 33.33, 27.43, 28.27, and 45.60 mm, respectively, proving that it produced the cellulase, protease, amylase, and lipase enzymes. The endophytic bacterial strain CO changed the color of the CAS medium from blue to purple, indicating its ability to produce a siderophores (Figure 8C–E). In the solid CAS medium, the zone diameter around the colony was 18.30 mm, while the siderophores production in the liquid CAS medium was 83.33%. The endophytic bacterium *P. poae* strain CO was tested for its ability to produce lipopeptides using MALDI-TOF mass spectrometry. As shown in (Figure 9), the mass spectra of *P. poae* strain CO revealed several prominent peaks ranging from *m*/*z* 1110.53 to 1483.65. The isolate produced molecules with mass peaks of 1100 to 1175 that correspond with cyclic lipopeptides of the viscosin family, including massetolides and viscosins [20], and molecules with mass peaks ranging from 1433 to 1483 that correspond to the lipopeptide fengycin A [21].

### 2.4. Improvement of Wheat Seedling Growth and Growth Promotion Properties

**Figure 10:**
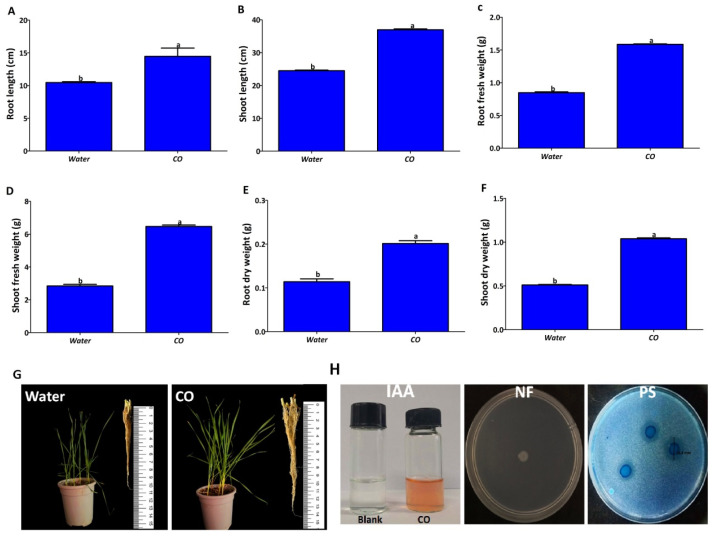
Effect of endophytic bacterium *P. poae* strain CO on the growth of wheat seedlings (**A**): root length; (**B**): shoot length; (**C**): root fresh weight; (**D**): shoot fresh weight. (**E**): root dry weight; (**F**): shoot dry weight; (**G**): seedlings treated and untreated with CO. (**H**): Plant growth properties assay: indole-3-acetic acid (IAA); nitrogen fixation (NF); and phosphate solubilization (PS). Data are mean value ± standard error of three replicates, and bars with the different letters are significantly different in LSD test (*p* < 0.05).

The effects of endophytic treatment on root length, shoot length, root fresh weight, shoot fresh weight, root dry weight, and shoot dry weight are shown in (Figure 10). Compared with the control (treated with water), the root length and shoot length of *P. poae* increased by about 33%, and the root fresh weight, shoot fresh weight, root dry weight, and shoot dry weight increased by about 50%. These results indicated that endophytic bacteria improved seedling growth in wheat seedlings compared to the control. The endophytic strain was tested for plant growth-promoting properties such as nitrogen fixation, IAA production, and phosphate solubilization. The measurement results in Figure 10H indicate that the endophyte CO strain developed a pink color in the presence of tryptophan, indicating the production of IAA with a concentration of 72.5 μg mL^−1^. In addition, nitrogen fixation ability was noted by observing their ability to grow on NFJ medium and dissolved phosphate in PVK agar medium at a rate of 15 mm through the clear zone surrounding the colony.

## 3. Discussion

In recent years, outbreaks of *Fusarium* seedling blight have become more serious. *Fusarium* blight control relies heavily on fungicides due to the scarcity of resistant varieties. However, the resistance of *F. graminearum* to chemical pesticides increases with excessive use, rendering many fungicides ineffective [22,23]. Moreover, since the fungus can infect wheat plants at different ages, from sowing to heading stage, farmers are faced with the difficulties of using fungicides to control this disease [24,25,26]. Therefore, there is an urgent need to develop new strategies for controlling FSB. In this study, we isolated the endophytic bacterium strain CO from garlic leaves, which showed a potent antifungal effect on *F. graminearum*. By analyzing the 16S rDNA sequence, the antagonistic endophytic bacterium strain CO isolated from garlic leaves in this experiment was identified as *P. poae*. Bacteria belonging to *P. poae* have been reported to possess strong biocontrol effects against several plant pathogens. For instance, strain RE*1-1-14 was reported to have the ability to inhibit sugar beet root rot caused by the soil-borne pathogen *Rhizoctonia solani* and *Verticillium wilt* of maize caused by *Verticillium nonalfalfae* and *V. dahlia* [27,28]. In addition, strains EE12 and JSU-Y1 have been shown to have antagonistic activity against *Fusarium oxysporum* and *Penicillium expansum* [29,30], while strain FL10F had antagonistic activity against *E. amylovora* [31]. Likewise, the results of the present study also support that the endophytic bacterium *P. poae* has an intensely antagonistic impact against FSB disease, which makes it a valuable resource for developing biocontrol agents. To further confirm the antagonistic effects of CO on wheat FSB disease, we performed an in-greenhouse test on wheat seedlings. The results showed that the CO application significantly reduced the FSB disease. Seed germination, for example, increased significantly to more than 50. This is strong evidence of the biocontrol potential of CO to control FSB disease. In agreement with our results, the endophytic bacteria *Bacillus amyloliquefaciens* stain XS-2, *Bacillus mojavensis* strain RRC 101, and endophytic fungus *Metarhizium anisopliae* were able to suppress the disease severity of FHB under greenhouse conditions [26,32,33].

Directly or indirectly, microbial biocontrol plays a vital role in suppressing fungal pathogens that infect plants. Direct results include antibiotic production, hydrolytic enzymes, and nutrient competition. Hydrolytic enzymes are one of the most effective mechanisms for inhibiting fungal pathogens, the most important of which are lipases, chitinases, amylases, proteases, and cellulases [15,34,35]. In agreement with these results, the endophytic bacterium *P. poae* was capable of producing amylases, lipases, proteases, and cellulases. In addition, *Pseudomonas* spp. produces siderophores with the ability to sequester Fe^3+^ in the rhizosphere, rendering this nutrient unavailable to phytopathogens (Masum et al. 2018). The ability of the endophytic bacterium *P. poae* to produce siderophores supports this conclusion. Lipopeptides (LPs) are secondary metabolites and amphiphilic molecules composed of a hydrophilic peptide group and hydrophobic fatty acid chains. Lipopeptides exhibit bactericidal and fungicidal activity by inhibiting cell wall synthesis or pores formed in the membranes of target microorganisms [28]. Cyclic lipopeptides from *Pseudomonas* spp. are intensively studied as biocontrol agents for plant disease management in agriculture [27,36,37] The endophytic bacterium *P. poae* produced lipopeptides corresponding with cyclic lipopeptides, such as massetolides, viscosins, and fengycin A. 

On the other hand, the endophytic bacterium *P. poae* was able to improve the growth of wheat plants. Various pathways of endophytic bacteria, such as phosphate solubilization, IAA production, nitrogen fixation, HCN production, and production of siderophores, can stimulate plant growth [14,16,38]. Although phosphorus is essential for plant growth, it is often present in low levels and difficult to absorb in agricultural soils. However, the endophytic bacteria can solubilize phosphate and make it easy for absorption by plants [39,40]. IAA is a plant hormone that plays an important role in cell enlargement, division, and root mimicry [41]. It is reported to be produced not only by plants but also by microorganisms [42]. Plant biomass, root length, and root surface area were enhanced by endophytic bacteria due to IAA production [18]. Nitrogen is an important factor for plant growth under different environmental conditions. Although the atmosphere is rich in nitrogen (78%), it cannot be used for metabolism and growth. It must be reduced to ammonia by any organism for use by a process called nitrogen fixation [43]. The ability of nitrogen fixation by endophytic bacteria is better than that by rhizospheric bacteria because they fix nitrogen directly within the plant with no loss compared to their counterpart [44]. In this study, the isolated endophytic bacterium had nitrogen-fixation ability, produced IAA, and solubilized phosphate, which contributed to the growth promotion observed in wheat. Despite all the advantages that endophyte bacteria confer on plants by protecting them from diseases and promoting their growth, some implementation challenges need to be considered. For example, host plants and environmental conditions can strongly influence endophyte use. In addition, the host plant’s age, geographic location, soil type, genotype, and even colonizing tissue can all play a big part in determining the type of endophytes it harbors. 

## 4. Materials and Methods

### 4.1. Isolation of Endophytic Bacteria

Entophytic bacteria were isolated from healthy garlic plants (*Allium sativum*) collected from five different locations in Hangzhou, China, according to the method of an earlier paper [45] with some modifications. In brief, for every location, the leaves and roots of five garlic plants were cut into small pieces (0.5 cm–1 cm) and disinfected with alcohol (70%) for 2 min, followed by sodium hypochlorite (1%) for 10 min, and then rinsed three times with sterile water under sterile conditions. For isolation, one gram of each sterile tissue was individually crushed in 9 mL of saline water (0.85% NaCl), subsequently followed by serial dilution up to 10^−5^. For each of these dilutions, 0.1 mL was spread on nutrient agar (NA) medium and then incubated at 30 °C for 2 days. The colonies were purified by transferring single colonies to a new NA plate. The isolated strains were stored in 30% (*v*/*v*) glycerol at −80 °C until use.

### 4.2. Identification of Endophytic Bacterium

The isolated endophytic bacterium was identified through 16S rRNA gene sequence analysis, according to [46] with some modifications. The CO isolate was grown for 24 h on NA medium, and a single colony was transferred to nutrient broth (NB) and incubated in a shaker at 30 °C overnight. The bacterial DNA from CO was isolated using the genomic bacterial DNA isolation Kit (Sangon Biotech (Shanghai) Co., Ltd., Shanghai, China) following the instructions in the protocol. The 16S rRNA gene was amplified using bacteria-specific primer pairs 27F (AGAGTTTGATCCTGGCTCAG) and 1492F (GGTTACCTTGTTACGACTT). To perform PCR amplification in 50 µL total volumes with 2 × TSINGKE Master Mix (TsingKe Biological Technology, Beijing, China), a Bioer XP Thermal Cycler (Hangzhou Bioer Tech. Co., Ltd., Hangzhou, China) was used. The thermal cycling parameters were as follows: an initial denaturation step at 95 °C for 5 min was followed by 30 cycles, with each cycle consisting of denaturing at 94 °C for 30 s, annealing at 53 °C for 30 s, and extension at 72 °C for 1 min/kb. The final extension at 72 °C for 5 min was used. The PCR amplicons were confirmed by agarose gel electrophoresis (1%) (*w*/*v*) and then purified using the StarPrep Gel Extraction Kit (GeneStar, Beijing, China). Purified PCR products were submitted to TsingKe Biological Technology, Beijing, China, for sequencing. Sequences from the 16S rRNA genes were aligned against a reference database using the NCBI blast program (https://www.ncbi.nlm.nih.gov/BLAST accessed on 28 December 2022). A phylogenetic tree was generated using the neighbor-joining method in the Mega 6.0 program and the sequence was deposited in the NCBI database.

### 4.3. In Vitro Antifungal Activity Assay 

#### 4.3.1. Inhibition of Mycelium Growth in Solid Medium

The diffusion agar method on potato dextrose agar (PDA) plates was used to test the antifungal activity of the endophytic bacterium *P. poae* strain CO against *F. graminearum* strain PH-1 obtained from the Institute of Biotechnology, Zhejiang University. In brief, a 5 mm disk was taken from a 5-day-old fungus and placed near the edge of the dish containing PDA media. At the edge of the other plates, a 5 μL culture of the *P. poea* strain CO (approximately 1 × 10^8^ CFU/mL), which had been grown on NB medium, was added. The PDA plates containing a fungus disc without *P. poae* strain CO served as the control. All the plates were incubated at 28 °C for 5 days, and the inhibition of mycelium growth was measured. The experiment was repeated three times with three replications.

#### 4.3.2. Inhibition of Mycelium Growth in Liquid Culture

The antifungal activity of the endophytic bacterium *P. poae* strain CO against PH-1 in liquid culture was assayed according to [47] with slight modification. Briefly, 1 mL of the endophytic bacterium (approximately ~1 × 10^8^ CFU/mL) culture, with a 10 mm disc taken from PH-1, was inoculated in 100 mL of potato dextrose broth (PDB), which was sterilized in a 250 mL conical flask, and incubated at 28 °C for 5 days. A conical flask of PDB containing a fungus disc without the endophytic bacteria was used as a control. The dry weight of the fungus culture grown with and without endophytic bacterium was measured, and mycelium growth inhibition was calculated.

#### 4.3.3. Cell Wall Morphology

According to [47], scanning electron microscopy (SEM) and transmission electron microscopy (TEM) were used to observe the effects of *P. poae* strain CO on the hyphal morphology of PH-1. In a nutshell, PH-1 hyphal samples were collected from the periphery of culture plates that were both incubated with and without *P. poae* strain CO. With the aid of SEM (TM10000, Hitachi, Japan) and TEM (JEM123030, JEOL, Akishima, Japan), the abnormalities of fungal hyphae were examined.

### 4.4. Antifungal Activity of Endophytic Bacterial Metabolites against Strain PH-1

#### 4.4.1. Preparation of Cell-Free Supernatants

*P. poae* strain CO cell-free supernatants (CFSs) were prepared using the method described in [48], with minor modifications. In brief, the CO strain was grown in NB medium at 30 °C with shaking overnight. A 1 mL of *P. poae* strain CO culture was transferred to NB medium in a 200 mL flask and incubated at 30 °C with shaking (200 rpm) for 3 days. The liquid cultures of *P. poae* strain CO (approximately 1 × 10^8^ CFU/mL) were centrifuged at 10,000 rpm and 4 °C for 10 min and filtered twice through 0.22 μm filters. A 100 μL of CFSs was spread out on an NA medium for one day to rule out potential contamination. 

#### 4.4.2. Effect of CFSs of *P. poae* on Mycelium Growth

The impact of CFSs of *P. poae* strain CO on the mycelium growth of PH-1 was evaluated according to [49], with slight modification. Briefly, CFSs were mixed with PDA medium at final concentrations of 20%, 30%, 40%, and 50% *v*/*v*. Plates without CFSs were scored as controls. The plates were inoculated with a fungal plug (5 mm in diameter) of PH-1, which was placed at the center. Inoculated plates were incubated at 28 °C for 5 days, and fungal diameters were recorded to calculate the antifungal effect. The experiment was repeated twice, with three replicates for each concentration.

#### 4.4.3. Inhibition of Fungal Colony Number 

The effect of four concentrations of CFS (20%, 30%, 40%, and 50% *v*/*v*) on colony numbers was assayed according to [50] with slight modifications. A total of 500 µL of conidia suspension and different concentrations of CFSs were mixed to a final volume of 2 mL. Tubes of conidia suspension mixed with water were recorded as controls. All tubes were incubated at 28 °C for 24 h. Of each concentration, 100 µL was spread on PDA and incubated at 28 °C for 3 days. The number of colonies formed on the plates were counted to determine the inhibition of the number of colonies in the treatment. 

#### 4.4.4. Effect of CFS on Spore Germination and Length of Germ Tubes

The effect of four concentrations of CFS (20%, 30%, 40%, and 50% *v*/*v*) on spore growth and germ tube length of the *F. graminearum* strain PH-1 was determined according to the method of [51] with some modifications. Briefly, 100 μL of a spore suspension (1 × 10^6^ spores per mL), prepared as described by [52], were mixed with an equal volume of CFSs in test tubes at a final concentration of 20%, 30%, 40%, and 50% *v*/*v*. Test tubes containing equal volumes of spore suspensions and ddH_2_O were used as controls. Then, 50 μL of the mixture was transferred to concave plates and incubated in the dark at 28 °C for 7 h. Spore growth rate and germ tube length were recorded using a light microscope. Each treatment consisted of five replicates, and the experiment was repeated twice.

#### 4.4.5. Effect of CFSs on Deoxynivalenol Production

Deoxynivalenol (DON) inhibition of *F. graminearum* strain PH-1 by CFSs was evaluated according to the method of [51] with minor modifications. Briefly, 100 mL of GYEP medium containing the indicated concentrations (20%, 30%, 40%, and 50% *v*/*v*) of CFSs was inoculated with 1 mL of spore suspension (1 × 10^6^ spores/mL) of *F. graminearum*. Flasks of GYEP medium without CFSs inoculated with the spore suspension were used as control. After seven days of incubation at 28 °C and 175 rpm, the supernatants were collected to determine DON production using a DON Plate ELISA Kit (Shanghai Yijishiye, Zhenjiang, China), and DON inhibition was calculated.

### 4.5. Seedling Protection from FSB

The wheat seedlings protection from FSB infection by *P. poae* was assayed using seed, coleoptile, and leaf treatments according to the methods of [53,54,55] with some modifications. For seed treatment, the seeds of the wheat cv. Jimai 22 were soaked in a 5% sodium hypochlorite solution for 20 min and then washed five times with sterile water to ensure surface sterility. Bacterial cells from the *P. poae* strain CO were collected and washed after 48 h of growth in NB medium by centrifugation at 12,000 rpm for 15 min. Cells were set again at 1 × 10^8^ CFU/mL in sterile distilled water. Sterilized seeds were soaked in a bacterial inoculum suspension for 4 h and then air-dried. Seeds inoculated with bacteria were sown in sterile soil at the rate of five seeds per pot, and 5 mL of macroconidia (1 × 10^6^ spores per mL) were added. Positive control groups consisted of uninoculated seeds grown in sterile soil with macroconidia, while the control group consisted of uninoculated seeds grown in sterile soil without macroconidia. All the pots were incubated in the growth chamber at 25 °C with 90% relative humidity and a 14 h/10 h photoperiod. Seed germination (%), pre-emergence damping-off (%), post-emergence damping-off (%), root length (cm), shoot length (cm), fresh weight (g), dry weight (g), seedling length vigor index, and seedling weight vigor index were all measured within 20 days of sowing. For coleoptile treatment, after three days of seed germination in hydroponic boxes at 20 °C, the tops (3 mm) were removed from the coleoptiles, which were then inoculated with 2 μL of spore suspension (1 × 10^6^ spores per mL) mixed with an equal volume of *P. poae* culture (1 × 10^8^ CFU/mL). The hydroponic boxes were incubated in a growth chamber at 28 °C with 90% relative humidity and a photoperiod of 14 h/10 h. The positive control was inoculated with Fg, while the control group was inoculated with water. After seven days post-inoculation (dpi), the seedlings were examined, and lesion size was recorded. For the leaf treatment, 25-day-old wheat seedlings were sprayed with a conidia suspension of F. *graminearum* (1 × 10^6^ spores per mL) mixed with an equal volume of *P. poae* culture (1 × 10^8^ CFU/mL) on both sides of the leaves by airbrush. Inoculated plants were incubated in a growth chamber at 28 °C with 90% relative humidity and a 14 h/10 h photoperiod. The control group was sprayed with sterile distilled water, while the positive control group was sprayed with Fg. The disease severity was determined as the percentage of seedlings with evident necrotic lesions.

### 4.6. Antifungal Properties of Endophytic Bacterium

#### 4.6.1. Protease Activity

Protease production of the endophytic bacterium was measured according to [47]. Fresh cultures of *P. poae* were added to Petri dishes, contented with skim milk agar medium (15 g skim milk, 0.5 g yeast extract, and 9.5 g of agar per liter). After two days of incubation at 30 °C, the appearance of a clear zone surrounding the bacterial colonies is a guide for protease production.

#### 4.6.2. Amylase Activity

Amylase production of the endophytic bacterium was measured according to [56]. Fresh cultures of *P. poae* strain CO were spotted into Petri dishes, contented with starch-agar medium (0.2% soluble starch and 1.5% agar), and incubated at 30 °C for 2 days. Then, the inoculated plates were immersed in a 1% iodine solution for 5 min. A clear area visible around the colonies indicates amylase activity. 

#### 4.6.3. Cellulolytic Activity

According to [47], the ability of an endophytic bacterium to produce cellulose was tested using carboxymethyl cellulose (CMC) agar medium (0.5 g CMC, 0.1 g NaNO_3_, 0.1 g K_2_HPO_4_, 0.1 g KCl, 0.05 g MgSO_4_, 0.05 g yeast extract, 0.1 g glucose, and 7 g agar for 1 L). In brief, *P. poae* strain CO was grown on a CMC medium and incubated at 30 °C for one week. Cellulase production was indicated by visible clear zones around colonies, which were confirmed on the plates after 5 min of flooding with 0.1% Congo red solution (1 M NaCl).

#### 4.6.4. Lipolytic Activity

According to [57], the ability of an endophytic bacterium to produce lipase was tested. The endophytic bacterium was added to Petri dishes filled with basal agar medium (10 g peptone, 5 g NaCl, 0.1 g CaCl_2_H_2_O, and 9.0 g agar per liter) supplemented with Tween 80, and incubated at 30 °C for one week. Visible dark and opaque zones around colonies indicate positive lipase production.

#### 4.6.5. Siderophores Assay

The endophytic bacterium’s ability to produce siderophores was tested according to the methods of [46], with some modifications. Briefly, the supernatant of *P. poae* was obtained from the culture grown in an MSA medium at 30 °C for two days and mixed with an equal volume of CAS solution. After 3 h of incubation in the dark at room temperature, a color change from blue to yellow, orange, or purple, signifies siderophore production. In addition, a colony of the endophytic bacterium was grown on the surface of the CAS agar medium and incubated at room temperature for three days. Siderophore production was recorded by the change in color from blue to orange, yellow, or purple around the colony. All treatments were repeated three times. 

#### 4.6.6. Detection of Lipopeptides Produced from CO Cells

Matrix-assisted laser desorption ionization–time of flight mass spectrometry (MALDI-TOF-MS), as previously described [46], was used to identify the lipopeptides produced by a colony of strain CO.

### 4.7. Improve Seedling Growth and Plant Growth-Promoting Properties

The effect of endophytic bacteria on wheat seedlings’ growth was assessed according to the method of [58], with slight modification. Following the disinfection with 2% sodium hypochlorite for 15 min and washing with distilled sterile water five times, seeds of wheat cv. Jimai 22 were soaked in P. *poae* (approximately ~1 × 10^8^ CFU/mL) for 4 h at 20 °C with constant shaking at 200 rpm, and were sown in pots of sterilized soil at a rate of six seeds per pot. The untreated seeds were used as control. Six replicates were applied for each treatment. The pots were incubated at 25 °C for 30 days, and root length, shoot length, root fresh weight, shoot fresh weight, root dry weight, and shoot dry weight were recorded. For plant growth-promoting properties, indole-3-acetic acid (IAA), phosphate solubilization, and nitrogen fixation were investigated. For IAA production, the ability of endophytic bacteria to produce IAA was estimated by growing the strain in King B medium modified with 0.1% (*w*/*v*) L-tryptophan and incubated at 30 °C for four days. The obtained supernatant was mixed with Salkowski’s staining reagent at a 2:1 ratio. IAA production was observed as a pink-red color development after 30 min of incubation in the dark, and the absorbance at 540 nm was measured using a SPECTRAmax^®^PLUS384 Microplate Spectrophotometer. The concentration of IAA in the supernatant was determined based on the IAA standard curve [59]. For phosphate solubilization, the capacity of the isolated strain to solubilize phosphate was evaluated by growing it on Pikovskaya’s (PVK) agar supplemented with K_2_HPO_4_ and CaCl_2_ [60]. Plates were incubated at 30 °C for seven days, and clear zones around the colonies indicated solubilized phosphate by the strain. The nitrogen fixation of the endophytic bacterium was determined, as described by [61], by inoculating the strain on nitrogen-free Jensens Medium. 

### 4.8. Statistical Analysis 

All experiments were performed in a completely randomized design, and the results were expressed as mean ± SD (standard deviation). Statistical analysis was performed using SPSS version 16.0 (SPSS Inc., Chicago, IL, USA). Differences between groups were calculated using test analyses. Results were statistically significant when the value was *p* < 0.05 or 0.01. 

## 5. Conclusions

In summary, the endophytic bacterium *P. poae* strain CO had a great capacity to control the phytopathogenic fungi, *F. graminearum*, and had the capacity for plant growth promotion. *P. poae* stain CO significantly suppressed *F. graminearum* in vitro and under greenhouse conditions. *P. poae* inhibited mycelium growth, colony number, spore germination, the germ tube length, and mycotoxin production in vitro, while the strain significantly reduced disease severity under greenhouse condition. To control *F. graminearum* and enhance the growth characteristics of wheat seedlings, *P. poae* exhibited the following properties: produced hydrolytic enzymes, siderophores, lipopeptides, and indole-3-acetic acid; solubilized phosphate; and fixed nitrogen. Finally, the endophytic bacterium demonstrated strong antagonistic properties in vitro and under greenhouse condition as well as a variety of plant growth-promoting properties. Thus, we suggest that it could be used as an alternate to synthetic chemicals and can serve as an effective method of protecting wheat from fungal infection while promoting plant growth. 

## Figures and Tables

**Table 1:** Disease assessment and growth parameters of seeds treated with endophytic bacterium *P. poae*.

Treatment	Disease Assessment and Growth Parameters								
	Sg (%)	Pre (%)	Post (%)	Rl (cm)	Sl (cm)	Fw (g)	Dw(g)	Slvi	Swvi
**Control**	93.33 ± 1.66 ^a^	6.66 ± 2.88 ^a^	0.00 ± 0.00 ^a^	5.27 ± 0.11 ^a^	8.90 ± 0.26 ^a^	0.82 ± 0.04 ^a^	0.19 ± 0.01 ^a^	1322.00 ± 7.22 ^a^	17.68 ± 0.35 ^a^
**Fg + CO**	70.00 ± 2.88 ^b^	30.00 ± 5.00 ^b^	7.10 ± 5.00 ^b^	3.50 ± 0.10 ^b^	4.90 ± 0.56 ^b^	0.55 ± 0.01 ^b^	0.08 ± 0.00 ^b^	602.67 ± 16.11 ^b^	5.24 ± 0.45 ^b^
**Fg**	15.00 ± 2.88 ^c^	85.00 ± 5.00 ^c^	80.33 ± 17.61 ^c^	1.47 ± 0.49 ^c^	2.00 ± 0.22 ^c^	0.21 ± 0.01 ^c^	0.03 ± 0.00 ^c^	52.97 ± 1.05 ^c^	0.35 ± 7.74 ^b^

Sg = Seed germination rates (%), Pre = pre-emergence damping-off (%), Post = post-emergence damping-off, Sl = shoot length (cm), Fw = fresh weight (g), Dw = dry weight (g), Slvi = seedling length vigor index), Swvi = seedling weight vigor index, CO = *P. poae* strain CO, and Fg = *F. graminearum.* Values with the different letters in each column are significantly different at (*p* < 0.05).

## Data Availability

The data is contained within the manuscript.
